# Open Reduction of Neglected Knee Dislocation: Case Report of a Rare Injury

**DOI:** 10.5704/MOJ.1611.011

**Published:** 2016-11

**Authors:** KC Kapil Mani, RC Dirgha Raj, Acharya Parimal, Pangeni Bandhu Ram

**Affiliations:** Department of Orthopaedics, Civil Service Hospital, Kathmandu, Nepal

**Keywords:** Arthrodesis, Open reduction, neglected knee dislocation, Steinman pins, total knee arthroplasty

## Abstract

Old neglected dislocation of knee joint is a rare injury. Any orthopaedic surgeon would have faced only a few cases of unreduced neglected dislocation in his life time practice. We report the case of a 30-year old male patient with one month old unreduced knee dislocation which was managed with open reduction and stabilization with two intra-articular crossed Steinman pins for six weeks, followed by removal of the pins and gradual weight bearing in hinged knee brace. At the end of one year, range of movement of knee joint was 0 to 50 degree with minimal knee pain on walking.

## Introduction

It is very unlikely that a single orthopaedic surgeon would have faced more than few cases of knee dislocation in his life time practice^1^. Neglected dislocation of knee joint is an even more uncommon event^2^. Because of rarity of the case, there is a paucity of reports in the literature and treatment options are also not clearly described^3^. We report the case of a one month old anterior knee dislocation managed by open reduction and stabilization with crossed Steinman pins followed by removal of pins and walking on the hinged knee brace six weeks later.

## Case Report

A 30 years old male patient attended our hospital with complaints of difficulty in walking, deformity, inability to flex the left knee joint and shortening of the limb for one month. He had been assaulted by unknown persons one month earlier and had sustained direct impact injury on the knee joint. There were no injuries on the other parts of body. He was able to walk only with the help of a crutch. There was no history of diabetes, hypertension, coronary artery disease, epilepsy or chronic respiratory problems. On examination there was distortion of shape of the knee joint, wasting of thigh muscle and shiny appearance of the skin. On palpation there was diffuse tenderness of the knee joint, proximal end of tibia was protruding anteriorly and patellar movement was not normal. There was no antero-posterior or varus-valgus laxity of the joint. Range of movement of knee joint was 0 to 20 degree, and shortening of the limb was 2.5cm. The distal neurovascular status however was normal. Radiograph confirmed anterior dislocation of the knee joint. Because of financial problems the patient had not sought any medical treatment for one month after the dislocation. He was not able to pay for the MRI, so we tested the status of ligaments intra-operatively. Both anterior and posterior cruciate ligaments were completely torn but the medial collateral ligament, lateral collateral ligament, medial meniscus, and lateral meniscus were intact. The knee joint was opened through a medial para-patellar approach; fibrous adhesions around the knee joint were released, blunt tip Homan’s retractor was inserted on the undersurface of the inter-condylar notch and by levering the retractor on the tibia, the joint was gradually reduced. After reduction, the joint was stabilized with two intra-articular crossed Steinman pins passed through the medial and lateral surface of the tibia. The knee joint was stabilized with a posterior POP slab for six weeks. Steinman pins were removed after six weeks and the patient was trained for partial weight bearing with a hinged knee brace locked in extension. At the end of one year the range of motion of knee joint was 0 to 50 degree; however the patient experienced mild pain on walking. Our plan was to perform either arthrodesis or total knee arthroplasty if the patient continued to have significant knee pain in future.

## Discussion

Knee dislocations are rare injuries^3^. Most acute knee dislocations are either reduced spontaneously at the accident site or reduced in the emergency department under sedation by closed manipulation^4^. There are case reports of old neglected knee dislocations ranging from four weeks to 15 years^2^. The treatment options include open reduction and fixation with Steinman pins, Ilizarov techniques, hinged external fixators, arthrodesis, and total knee arthroplasty^5^. Vincente-Guillen *et al*^2^ reported a 15-year old posterior knee dislocation treated with open reduction and external fixation, progressive fixation and finally arthrodesis of the joint. Petrie *et al*^5^ reported two cases of 4-month old knee dislocations successfully managed with total knee arthroplasty with constrained prosthesis.

**Fig. 1:**
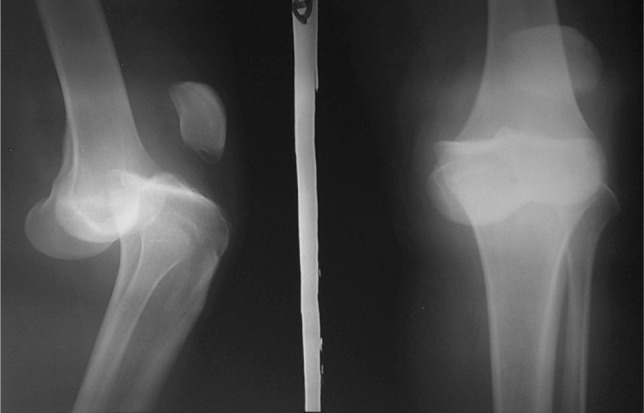
Radiographs of knee joint in antero-posterior and lateral views showing dislocated knee joint.

**Fig. 2:**
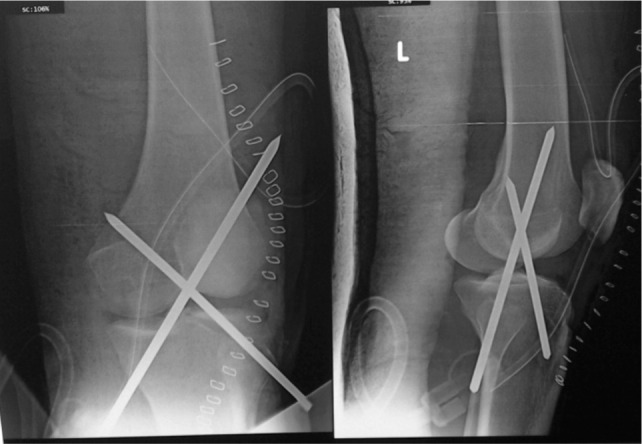
Antero-posterior and lateral view of radiographs showing reduction of joint and fixation with two crossed Steinman pins

**Fig. 3:**
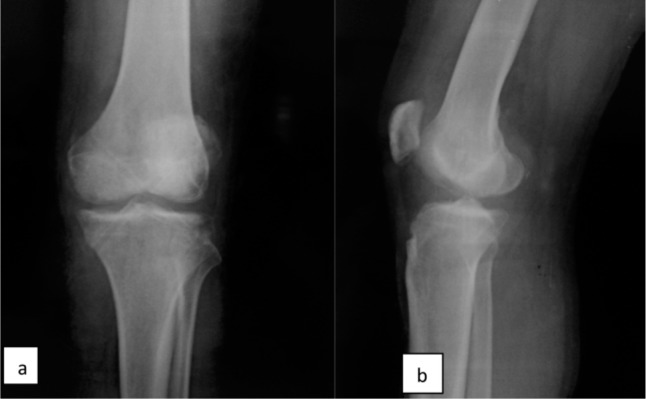
(a) Antero-posterior and (b) lateral radiographs of knee joint showing reduction of joint after removal of Steinman pins.

The purpose of treatment was to achieve a painless, mobile and stable knee joint without much functional disabilities^3^. Since the duration of knee dislocation in our case was only four weeks, we open reduced the joint and maintained with two crossed Steinman pins. The joint was maintained in the reduced position even after removal of Steinman pins and some degree of movement was possible. This way we were able to avoid the much complicated procedures like arthrodesis and total knee arthroplasty. Many patients refuse the arthrodesis due to loss of knee movement and inability to pay for TKA because of financial problems. In conclusion, neglected knee dislocations are rare. Open reduction and fixation with Steinman pins followed by gradual weight bearing in hinged knee brace is a good early option and to delay the much complicated surgeries like arthrodesis or TKA if needed in the future.
